# An Anatomical Description of the Human Gravid Uterus and Its Contents

**Published:** 1844-04

**Authors:** 


Art. IX.—*An Anatomical Description of the Human Gravid Uterus and its contents. By the late W. Hunter,* m.d. &c. Second Edition. By E. Rigby, m.d. &c.—*London*, 1843. 8vo, pp. 76. Eight Plates.

This is a seasonable republication of a classical work that had become very scarce; and the profession is under an obligation to Dr. Rigby for having undertaken the task of reediting it. The plan of the present publication is precisely such as we think most proper in like cases. The original work is reprinted, and only such notes—few in number—added as seemed to the editor absolutely necessary to the clearness or just appreciation of the author's statements at the present time. In one of his notes to the section on the Nerves of the Uterus, the editor, whilst acknowledging, in strong terms, the claims of Dr. Lee as an anatomical inquirer, in our opinion falls short of doing full justice to this physician's merits as regards his researches on the nerves of the gravid uterus. Surely, after the publication of Dr. Lee's work on the subject, Dr. Rigby ought not to have stated that Dr. Hunter's treatise "still contains the chief, if not all, of what we yet know for certain about the nerves of the gravid uterus." But as we have already noticed this subject, we shall not further refer to it on the present occasion. Taken as a whole, Dr. Rigby's notes are excellent; and he has creditably discharged his task as editor. In looking over the text we have observed several errors of the press which a vigilant printer ought to have avoided; but these detract, in no respect, from the sterling merit of the work, which we recommend to the attention of every anatomist, and every practitioner of midwifery.

